# Effects of Counter-Stereotypes Cognitive Training on Aging Stereotypes in 12– to 13-Year Olds

**DOI:** 10.3389/fpsyg.2021.693979

**Published:** 2021-10-15

**Authors:** Li Chen, Xiaohuan Zhang, Shuaishuai Fan, Lezhen Fu, Jiaojiao Zhao

**Affiliations:** School of Psychology, Northwest Normal University, Lanzhou, China

**Keywords:** aging stereotypes, evaluative conditioning technique, teenager, counter-stereotypes, counter-stereotypes scenario

## Abstract

The purpose of this study was to investigate the effect of counter-stereotypes cognitive training on adolescents’ aging stereotypes and to further investigate the best training method to intervene in aging stereotypes by comparing the effect of single and multiple intervention training methods on aging stereotypes and their retention effects. Three experiments examined the different intervention outcomes of different counter-stereotypes cognitive training on adolescent aging stereotypes. The study used a randomized block group experimental design and recruited a total of 183 middle school students for testing. Experiment 1 verified the effect of counter-stereotypes cognitive training by taking a single training task (evaluative conditioning technique), randomly assigning subjects to different conditions (training task or unrelated drawing task), and administering a follow-up test 24h after the posttest. Experiment 2a compared the effects of multiple versus single cognitive training, where we took multiple (adding the counter-stereotypes situational storytelling method) versus single training tasks and administered a follow-up test 72h after the posttest. Experiment 2b increased the number of training sessions based on Experiment 2a, with a second intervention training 72h after the end of the posttest and a follow-up test 72h after the second training. Experimental results suggest that evaluative conditioning techniques are effective in weakening subjects’ aging stereotypes, but are less effective in maintaining them. Compared to a single training task, multi-tasking is more effective and the effects of the intervention are maintained for up to a week by increasing the number of training sessions.

## Introduction

As the global older adult population continues to grow, population aging will be one of the major issues we face in this century (Cheng and Heller, 2010). Population aging not only hinders the development of the country in various fields, but also has many negative effects on older adults themselves, which have received active attention from researchers in different fields at home and abroad, and researchers have focused their attention on the study of older adults affected by age stereotypes. Our perceptions and expectations of older adults as a specific social group are the aging stereotype (Levy et al., 2000b). A typical and strong stereotype in Western culture is the negative aging stereotype. Research on aging stereotypes first originated in the 1950s, and with the acceleration of global aging, research on aging stereotypes began to enter a boom in the 1980s to 1990s in the West (He et al., 2013), and its research on aging stereotypes was more adequate. Little research has been done on aging stereotypes in China, but more on aging attitudes associated with aging stereotypes (He et al., 2013), and it has mainly focused on status surveys and the development of measurement instruments. However, population aging is closely related to national politics, economy, and social construction, and as China’s population ages, the Chinese government has elevated it to the level of a national strategy. At the same time, respect for older adults is a traditional Chinese virtue, which has led society to give them some special care, but the potential aging stereotype that comes with this special care also pushes them to the margins of society and has a negative impact on their bodies and minds. Therefore, the study of aging stereotypes, especially the negative effects caused by aging stereotypes, has received increasing attention from Chinese scholars (Li, 2010). Negative aging stereotypes as a social cognitive factor have a negative impact on older adults that cannot be ignored. Research has found that aging stereotypes have a significant impact on people’s physiology, cognition, behavior, and daily life (He et al., 2013; Bae et al., 2018; Chiviacowsky et al., 2018; Li and Xu, 2018). For example, aging stereotypes can affect individuals’ physiological functions, such as blood pressure, hearing (Levy et al., 2000a, 2009), cognitive functions such as memory performance and judgment (Wheeler and Petty, 2001; O’Brien and Hummert, 2006; Radvansky et al., 2010), and everyday habits such as writing and reading, and consumption behavior (Bargh et al., 1996; Levy et al., 2000a; Bae et al., 2018).

With the deepening and expansion of stereotype research by domestic and foreign researchers, research on counter-stereotypes has also been actively developed. Counter-stereotypes are traits exhibited in group members that are inconsistent with or contrary to stereotypes (Garciamarques and Mackie, 1999; Santos et al., 2012), as well as backgrounds that do not match each other (Wittenbrink et al., 2001; Casper, 2010). Among them, counter-stereotypes cognitive training plays a key role in reducing the influence of negative stereotypes. Counter-stereotypes cognitive training has been shown to be effective in diminishing or inhibiting the activation and application of stereotypes across the domains of race, occupation, and gender (Marini et al., 2012; Meijs et al., 2015; Lai et al., 2016; Burns et al., 2017). Although aging stereotypes have not been given corresponding attention in academia (Barber, 2017), aging stereotypes as a socio-cognitive factor, it is reasonable to believe that counter-stereotypes cognitive intervention training may have the same effect in suppressing aging stereotypes and eliminating the negative effects of aging stereotypes on older adults. Lai et al. (2016) found that the counter-stereotypes sample exposure method, counter-stereotypes situational story method, feedback on false implicit-association test (IAT) results, and evaluative conditioning technique were all effective interventions by comparing different counter-stereotypes intervention training methods. Among them, the evaluative conditioning technique and the counter-stereotypes situational storytelling method are easier to operate and maintain the training effect for a longer period. Counter-stereotypes storytelling is an effective way to weaken or eliminate stereotypes by exposing subjects to a variety of typical counter-stereotypes situations. It has been shown that targets are made to exhibit behaviors inconsistent with stereotypes through false story scenarios, news reports, or people’s subjective accounts, whether it is a plant-related situation (flowers are dangerous, and insects are safe; Foroni and Mayr, 2005; Lai et al., 2014) or a human-related situation (e.g., black heroes and white villains), which can develop distinct counter-stereotypes through stories (Dasgupta and Greenwald, 2001; Marini et al., 2012; Burns et al., 2017). Some researchers have presented subjects with a compiled report of an assault by using a false story scenario or report. The content of this incident is different from what is usually known, in which the black people in the story are the rescuers and the white people are the attackers (Dasgupta and Greenwald, 2001; Marini et al., 2012). The results were found to be consistent with those of previous studies, indicating that the counter-stereotypes situational storytelling method can effectively inhibit the activation of stereotypes. Counter-stereotypes situational storytelling is a method that uses vivid story scenarios or videos to stimulate the subjects’ thinking and thus inhibit the activation of stereotypes. This method applies to a variety of vivid situations, such as racial stereotypes and age stereotypes. However, this method has strict requirements for the selection and evaluation of story materials.

The evaluative conditioning technique is a method discovered and named by (Martin and Levey, 1978), which focuses on making a relevant connection between two things through multiple pairwise evaluations and transferring attitudes, emotions, etc., about one thing to the other. The associative-propositional evaluation model summarized by Gawronski and Bodenhausen (2006) found that the typical method of changing implicit attitudes is a progressive change in social structure achieved through the action of evaluative conditioning (Burns et al., 2017). For example, Dijksterhuis (2004) used an evaluative conditioning technique in which subjects repeatedly paired their self-represented words with positive words and found that their implicit self-esteem was effectively increased after repeated practice. Other researchers have found that having pairs of subjects repeatedly paired with pictures of faces (black faces and white faces) and stimulus potency words (positive and negative) can be effective in changing subjects’ internalized biases and stereotypes through multiple exercises (De et al., 2001; Olson and Fazio, 2001, 2002, 2006; Burns et al., 2017). The evaluative conditioning technique applies to a wide range of conditions and can be applied to a variety of stereotype intervention training such as race, gender, and age. The evaluative conditioning technique is simpler and more efficient than other intervention techniques. However, this technique also tends to produce practice effects in subjects.

However, comparing previous studies found that the effects of both counter-stereotypes cognitive intervention training had better effects in the short term; however, the effects of the intervention training were poorly maintained and the effects of conducting a single intervention training were only maintained for a few hours or days (Burns et al., 2017). In summary, we found that the training modalities used by researchers in previous studies were homogeneous, the number of intervention training sessions was limited, and few follow-up studies were conducted (Dasgupta and Greenwald, 2001; De et al., 2001; Marini et al., 2012; Burns et al., 2017), although the intervention training was effective but poorly maintained. From this, we can speculate that the reasons for the short-term effectiveness of the intervention training and the poor maintenance of the long-term effect may be due to the single mode of intervention training and the insufficient intensity and frequency of the intervention training. Therefore, whether enriching the intervention and increasing the intensity and frequency of the intervention training can achieve the counter-stereotypes effect to a greater extent will be one of the questions to be explored in this study.

In addition, the age of the subjects had an important effect on the intervention effect. Research on aging stereotypes suggests that aging stereotypes include not only stereotypes of older adults about themselves, but also stereotypes of older adults by groups other than older adults (Levy, 2003; Hess et al., 2004; Remedios et al., 2010). Previous research has shown that activation of stereotypes of aging in young people also affects young people’s performance in cognition (Zafeiriou and Gendolla, 2017) and that young people can achieve the same level of cognitive performance as older adults by mobilizing less energy in cognitive tasks compared to older adults (Smith and Hess, 2015). This is due to the high cognitive plasticity of young people and the ease with which formed biases, stereotypes, etc., can be changed (Bigler and Liben, 2006; Lai et al., 2014, 2016). Among these, childhood is a critical period of cognitive development and an important stage for changing stereotypes, where prejudice and stereotypes may be the easiest to change (Devine, 1989; Greenwald and Banaji, 1995; Rudman and Fairchild, 2004). In addition, evidence based on social cognitive development studies suggests that implicit bias may be more likely to change in older children and that this mechanism may better allow older children to change their evaluations of the group after exposure to counter-stereotypes (Lai et al., 2014). For example, Gonzalez et al. (2017) used the counter-stereotypes sample exposure method to expose children and adolescents aged 5–13years to a positive and positive Black sample and found that age differences had a significant impact on the intervention effects of the training, and while there was a reduction in children’s prejudice against black people, it was limited to adolescents aged 10 to 13years, the age group most prominently affected by the counter-stereotypes intervention training and where implicit prejudice was most likely to change.

In summary, by comparing previous studies, we found that the current counter-stereotypes cognitive intervention training has the following limitations: (1) The cognitive intervention training used is relatively homogeneous (2) intervention effects are poorly maintained and effective only in the short term (3) fewer interventions and fewer follow-up studies were conducted, and (4) subjects were selected mostly from adults and less from adolescents with high cognitive plasticity. Therefore, the controversies and shortcomings of the above training methods, training intensity, and age effects on the effect of counter-stereotypes intervention training are addressed. In this study, we selected junior high school students (12–13years old) with high social cognitive plasticity as subjects and used the evaluative conditioning technique and the counter-stereotypes situational storytelling method to enrich the intervention training, extend the duration of the intervention training, and increase the frequency of the intervention training, and to measure the retention effect of the counter-stereotypes cognitive intervention training through a follow-up study. Two hypotheses are proposed in this study: (1) Single-task intervention training (evaluative conditioning techniques) can be effective in weakening aging stereotypes in secondary school students and (2) compared to single-task intervention training, multiple training tasks are more effective and longer lasting in weakening aging stereotypes in secondary school students.

## Experiment 1: the Effect of a Single Training Task on Aging Stereotypes

Experiment 1 was conducted to better understand whether single-task intervention training can effectively weaken the aging stereotypes of middle school students and to further verify the results of previous intervention training research.

### Method

#### Participants

Seventy students (*M_age_*=12.31, *SD*=0.50) participated in Experiment 1. All participants were randomly recruited from three seventh-grade classes at a middle school in Zunyi, Guizhou province. Six participants failed to complete all the tests due to a computer glitch, and three participants in the pretest did not complete the experimental tracking. After D-value treatment, it was found that the response errors of the three participants exceeded 70%. Therefore, 58 effective samples were finally obtained (31 in the experimental group and 27 in the control group). All recruited participants had normal or corrected-to-normal vision, were proficient in using computers, and had not participated in a similar experiment before. All participants had parental consent before taking part in the experiment and were given a small gift at the end.

#### Materials

The experimental materials consisted mainly of IAT materials and intervention training materials. First, we selected conceptual and attribute words for older adults and younger adults by distributing a questionnaire (Appendix 1). The questionnaire was divided into three main parts. The first part asked the subjects to write conceptual words related to older adults and younger people. The second part required subjects to write at least five adjectives related to physical characteristics (describing the bodies of older adults and younger people), cognitive characteristics (about the cognitive abilities of younger and older adults), and personal expressions (about attitudes, mental states, etc.) of attributes related to older adults and younger people. The third part asked the subjects to write positive and negative words that describe the physical characteristics, cognitive characteristics, and personal expressions of the individuals. A total of 150 copies of vocabulary questionnaires were sent out, and 139 valid questionnaires were collected. The questionnaires were recovered after word frequency analysis and screening and combined with the vocabulary used in the measurement of age stereotypes by He et al. (2013), Li (2010), and Zuo et al. (2007). We ended up with the following findings:

1. Concept words: 15 for older adults and 15 for the young.
2. Attribute words: 46 attribute words describing physical characteristics of older adults and younger adults (22 for older adults and 24 for younger adults), 52 attribute words describing personal expressiveness (26 for older adults and 26 for younger adults), and 44 attribute words describing cognitive characteristics (20 for older adults and 24 for younger adults).
3. Positive or negative words: 49 positive or negative words describing physical characteristics of older adults and younger adults (23 positive and 26 negative), 45 positive or negative words describing cognitive characteristics (23 positive and 22 negative), and 64 positive or negative words describing personal expressiveness (32 positive and 32 negative).

We screened and organized the collected words and then recruited subjects again to perform a secondary evaluation of the screened words (Appendix 2). Considering that college students have higher knowledge and relatively mature cognition, they have a richer understanding of older adults and younger people and are more likely to judge and filter the vocabulary. Therefore, we recruited a total of 30 university students enrolled in the School of Psychology and selected relevant conceptual words, positive and negative words, and attribute words by analyzing the word frequency of the subjects’ vocabulary evaluation results and then ranking them according to the evaluation results in order. We ended up with the following findings:

1. 20 concept words: 10 for older adults and 10 for younger adults. Older adults concept words, such as crutches and wheelchairs, and younger adults concept words, such as games and staying up late.
2. 36 positive and negative words: 6 positive words describing physical characteristics of individuals, such as tall and fit; 6 negative words, such as clumsy and weak. Six positive words describing the individual’s cognitive abilities, for example, extremely intelligent and capable. Six negative words, such as unresponsive and clumsy. Six positive words describing personal expressiveness, for example, enthusiastic and clear. Six negative words, such as cranky and pessimistic.
3. 60 attribute words: 20 attribute words describing physical characteristics of older adults and younger adults, such as gray hair and robust, 20 attribute words describing cognitive characteristics, such as unresponsive and eloquent, and 20 attribute words describing personal expressiveness, such as nagging and cranky (See Appendix 3).

The measurement used in the present study was adopted from the classic IAT paradigm of Greenwald et al. (1998) and the IAT 7 (Block) paradigm used by He et al. (2013) and Li (2010). The experimental program was compiled using E-prime 2.0 (see Table 1).

**Table 1:** Flow chart of IAT test procedure.

Test program(Block)	Task Description	Function	Trial run	Operational tasks	Sample
Block 1	Judgment of age concept words	Practice	10	F: Old people’s things J: Youth thing	F: Crutch J: Basketball
Block 2	Age attribute word judgment	Practice	10	F: The older adults J: The young man	F: Grandmotherly J: Brave
Block 3	Compatible concept-attribute word joint judgment	Practice	30	F: Old people’s things-The older adults J: Youth thing-The young man	F:Crutch-Grandm-otherly J:Basketball-Brave
Block 4	Compatible concept-attribute word joint judgment	Test	120	F: Old people’s things-The older adults J: Youth thing-The young man	F:Crutch-Grandm-otherly J:Basketball-Brave
Block 5	Age attribute word judgment	Practice	10	F: The older adults J: The young man	F: Grandmotherly J: Brave
Block 6	Incompatible concept-attribute word joint judgment	Practice	60	F: Old people’s things-The young man J: Youth thing-The older adults	F: Crutch-BraveJ:Basketball-Gran-dmotherly
Block 7	Incompatible concept-attribute word joint judgment	Test	120	F: Old people’s things-The young man J: Youth thing-The older adults	F: Crutch-Brave J:Basketball-Gran-dmotherly

#### Design and Procedure

The experiment used a 2 (group: experimental group, control group)×3 (measurement time: pretest [IAT_1_], posttest [IAT_2_], and intervention effect tracking test [IAT_3_]) mixed design, and the dependent variable was the effect value (D) after the average reaction time before and after the IAT was converted *via* the Greenwald et al. (2003) method.

The participants were randomly assigned to either the experimental group or the control group, and the test time was arranged according to the different groups after the allocation. Each participant completed a practice experiment before starting the formal test, which lasted for 2min, and the formal test began after all the participants finished the practice experiment and confirmed they understood the experimental procedure.

**Figure 1:**
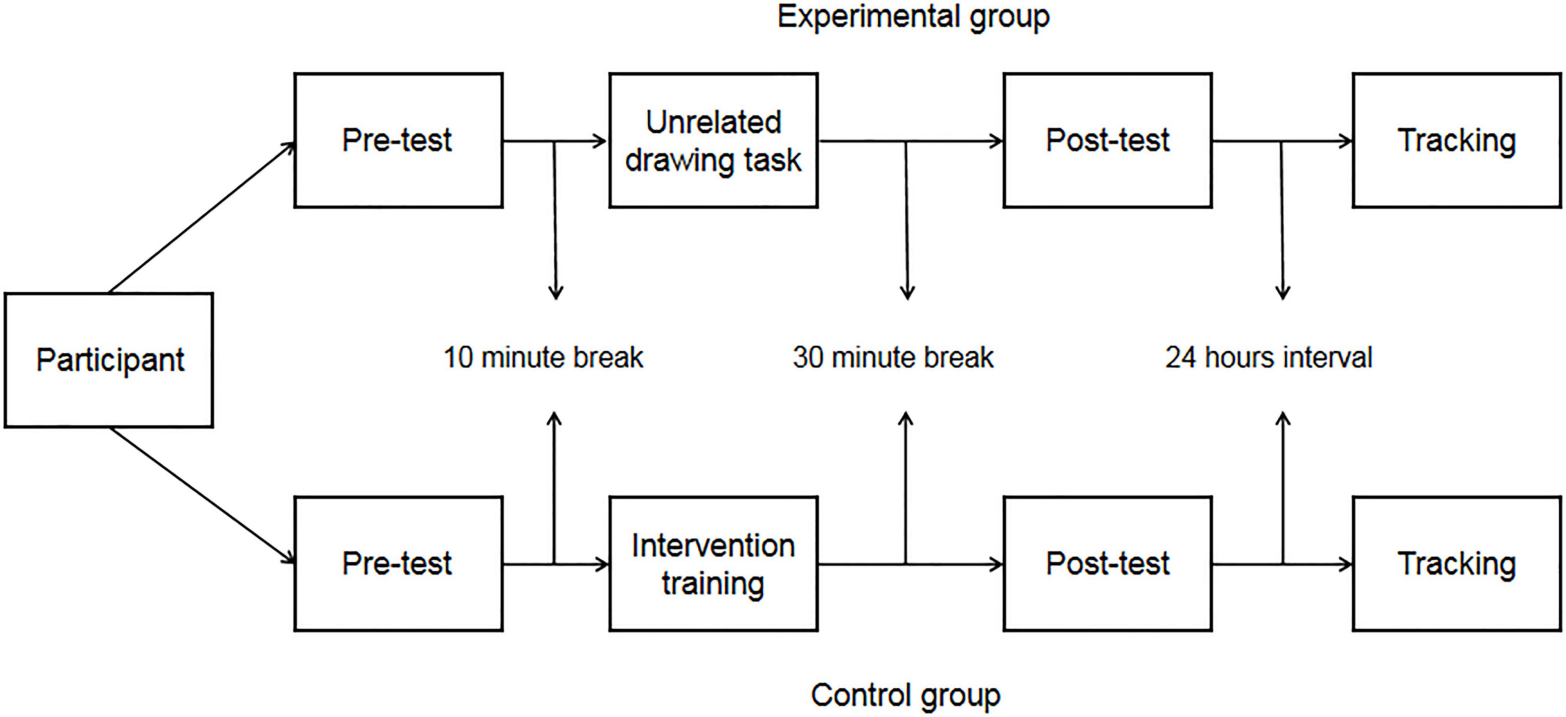
Experimental flow chart of Experiment 1.

**Figure 2:**
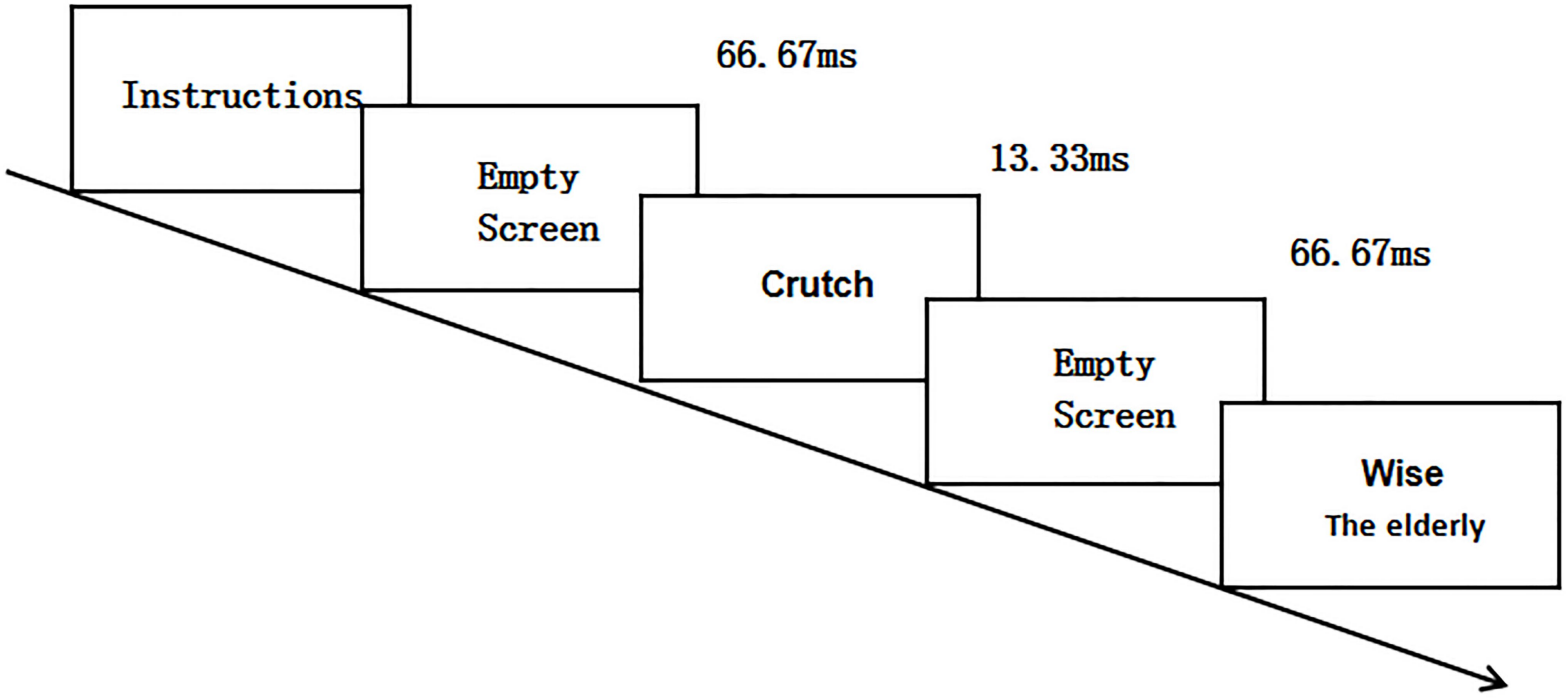
Intervention training flow chart.

During the formal test, the tasks performed by the experimental group and the control group were different. The test group was required to complete the pretest (IAT_1_), intervention training task, posttest (IAT_2_), and tracking test (IAT_3_). The control group was required to complete the pretest (IAT_1_), unrelated drawing task, posttest (IAT_2_), and tracking test (IAT_3_). The pretest confirmed the baseline value, and the interval of the tracking test was 24h. See Figure 1 for the specific schedule of the experiment. The intervention training task program was compiled using E-prime2.0, and the materials comprised the collected positive and negative words. Intervention training mainly combined the evaluative conditioned reflex technology adopted by Burns et al. (2017) and Li (2010). The procedure of the intervention was as follows: First, the instruction was presented in the center of the screen with a white background, and the subject understood the instruction and pressed the space bar, followed by a blank screen at 66.67ms. The blank screen disappeared, and then, the stimulus (young vocabulary (fashion) or old vocabulary (crutches)) was presented for 13.33ms without requiring the subject to respond to the stimulus. After the stimuli were presented, a blank screen was then presented for 66.67ms, and after the blank screen disappeared, positive words (wise, older adults) or negative words (cranky, younger adults) were presented, at which point the subjects were asked to categorize the words that appeared (by pressing the “F” key or the “J “key), positive attribute words (“F” key) negative attribute words (“J” key), intervention training for a total of two hundred trials of positive (wise, older adults) and negative (cranky, young adults) lexical matches, and the results of the training were not counted in the final statistical analysis. (See Figure 2).

After completing the training task, the experimental group rested for 30min before completing the posttest (IAT_2_) and continued with the follow-up test of training effects (IAT_3_) after an interval of 24h. After completing the IAT pretest, the control group took a 10-min break, after which they completed the painting task. After completing the drawing task, a 30-min break was taken, followed by a posttest (IAT_2_), and a 24-h interval followed by a follow-up test (IAT_3_).

#### Results and Analysis

Reactions measured by IAT were processed and converted by calculating the effect value (D) using the method proposed by Greenwald et al. (2003). Repeated measures analysis of variance (ANOVA) showed that the main effect of measurement time was significant, *F*(2, 112)=14.65, *p*<0.001, $\eta_{p}{}^{2}$ =0.21. The multiple comparison results showed that in the control group, the effect value of pretest and posttest was not significantly different, *t*(26)=1.08, *p*=0.29, *Cohen’s d*= 0.42 (*M_c-pre_*=0.73, *SD*=0.45, *M_c-post_*=0.61, *SD*=0.28). There was also no significant difference between the posttest effect value and tracking test effect value in the control group, *t*(26) =1.38, *p*=0.18, *Cohen’s d*= 0.54 (*M_c-post_*=0.61, *SD*=0.28, *M*_c-tra_=0.49, *SD*=0.32). However, the pretest effect value was significantly different from the tracking test effect value in the control group, *t*(26)=2.06, *p*=0.016, *Cohen’s d*= 0.81 (*M*_c-pre_=0.73, *SD*=0.45, *M*_c-tra_=0.49, *SD*=0.32). In the experimental group, there was a significant difference between the pretest and posttest effect values, *t*(30)=5.11, *p*<0.001, *Cohen’s d=1.87* (*M*_e-pre_=0.87, *SD*=0.47, *M*_e-post_=0.39, *SD*=0.26). There was no significant difference between the posttest and tracking effect values in the experimental group, *t*(30)=−1.56, *p*= 0.13, *Cohen’s d=−*0.57 (*M_e-pre_*=0.39, *SD*=0.26, *M*_*e*-tra_=0.48, *SD*=0.27). However, there was a significant difference between the pretest and tracking effect values in the experimental group, *t*(30)=3.66, *p*<0.001, *Cohen’s d=1.34* (*M*_e-pre_=0.87, *SD*=0.47, *M*_e-tra_=0.48, *SD*=0.27). The main effect of group was not significant, *F*(1, 56)=0.31, *p*=0.577, $\eta_{p}{}^{2}$ =0.006. (see Table 2).

**Table 2:** Mean IAT effect values (D) by group.

Group	Pretest *M(SD)*	Posttest *M(SD)*	Tracking *M(SD)*
Control group	0.73(0.45)	0.61(0.28)	0.49(0.32)
Experimental group	0.87(0.47)	0.39(0.31)	0.48(0.27)

^a^*N*=58.

**Figure 3:**
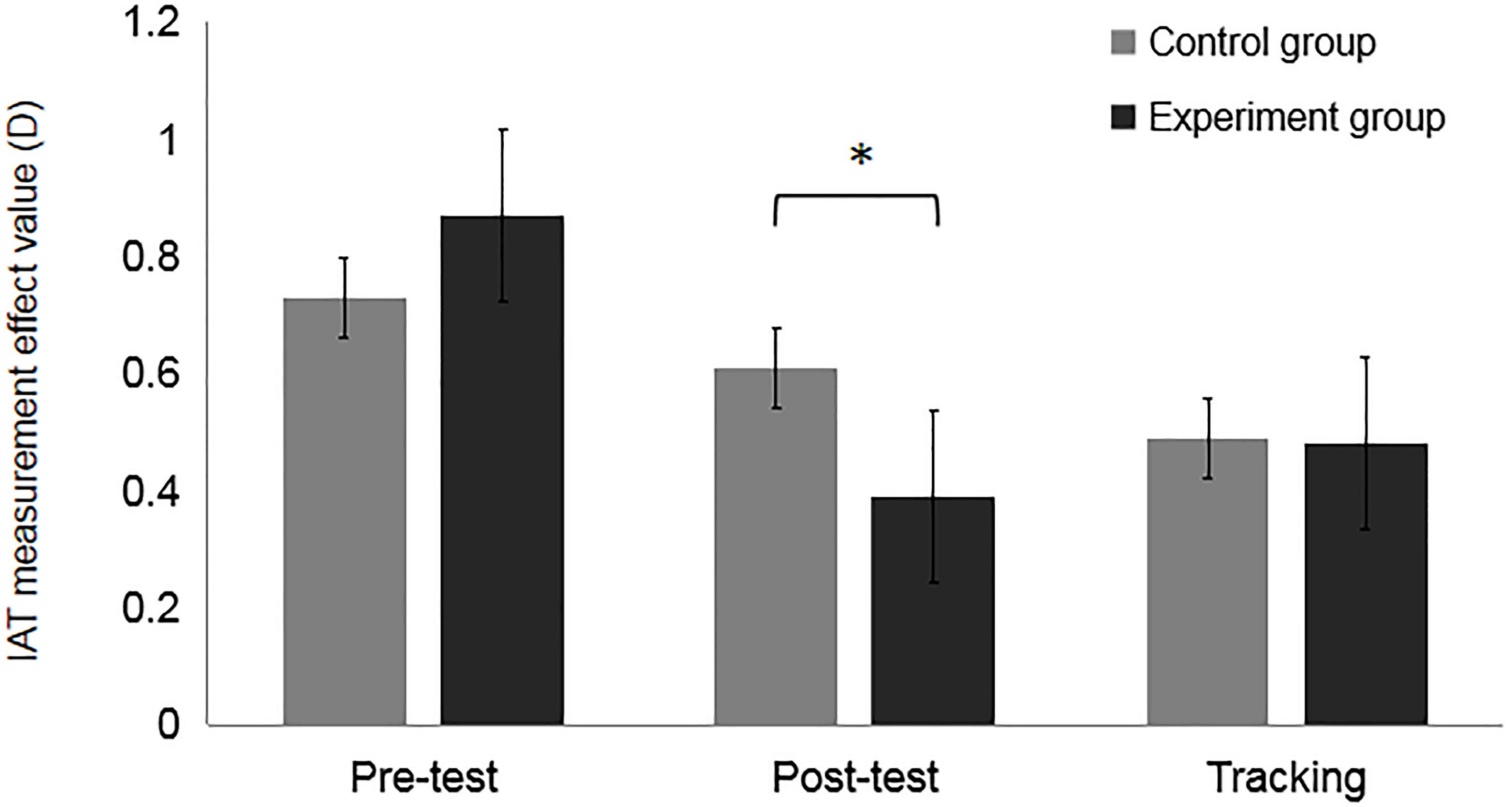
Results of the measurement of the effect value (D) of the training effect for different groups, *p<0.001.

The interaction between group and measurement time was significant, *F*(2, 112)=3.82, *p*=0.03, $\eta_{p}{}^{2}$ =0.07. Simple effects analysis showed that the pretest effect values of the experimental and control groups were larger than the pretest effect values of the control group, which indicated that the stereotypes were stronger in the experimental group than in the control group before the intervention training, but there was no too significant difference between the two groups of subjects, *F*(1,56) =1.34, *p* = 0.25, $\eta_{p}{}^{2}$ =0.023 (*M_e-pre_* =0.87, *SD* =0.47, *M_c-pre_* =0.73, *SD* =0.45). Compared with the control group, the posttest effect value of the experimental group was significantly lower *F*(1, 56)=9.49, *p*=0.003, $\eta_{p}{}^{2}$ =0.15 (*M*_e-post_=0.39, *SD*=0.26, *M*_c-post_=0.61, *SD*=0.28). This suggested that intervention training can effectively weaken aging stereotypes in participants. After 24h, tracking tests found that the effect value of the experimental group was not different from that of the control group, *F*(1,56)=0.02, *p*=0.88, $\eta_{p}{}^{2}$ =0.000 (*M*_*e*-tra_ =0.49, *SD* =0.32, *M*_c-tra_ =0.48, *SD* =0.27), which indicated that although the intervention training was effective, the effects remained poor and began to decline after 24h (see Figure 3).

### Discussion

The results of Experiment 1 replicated previous studies and proved that counter-stereotypes intervention training could effectively suppress aging stereotypes. Although the participants were selected differently and only underwent one intervention, the results of the intervention training were consistent with those of the adult participants in previous studies (Aboud, 2005; Baron, 2015; Lai et al., 2016; Burns et al., 2017). Although the effect of intervention training was maintained poorly and gradually declined after 24h, it did prove that the aging stereotype of the participants could be weakened to a certain extent.

## Experiment 2A: a Comparative Study of Multiple Training Tasks Versus Single Training Tasks

Experiment 2a was conducted to examine whether the dual intervention training tasks (adding counter-stereotypes scenario method) or a single intervention training task (evaluative conditioning technique) would have better effects over a longer period. Therefore, in Experiment 2a, we gave the experimental group dual training tasks and the control group a single training task and then determined which group showed better retention of training effects when performing different tasks.

### Method

#### Participants

Seventy students (*M_age_*=12.28, *SD*=0.45) participated in Experiment 2a. All participants were randomly recruited from the seventh grade of a middle school in Zunyi, Guizhou province, and were 12–13years old. Two participants did not complete all the experimental tests due to a computer error, and two participants in the pretest did not complete the tracking test. After D-value treatment, it was found that the response errors of the two participants exceeded 70%; therefore, 64 effective samples were finally obtained (34 in the experimental group and 30 in the control group). All recruited participants had normal or corrected-to-normal vision, were proficient in using computers, and had not participated in a similar experiment before. All participants volunteered and were given a small gift at the end of the experiment.

#### Materials

Both the experimental and control groups were required to complete two tasks, where the experimental group was required to complete a training task (evaluative conditioning technique) and a video viewing task (counter-stereotypes situational story), and the control group was required to complete a training task (evaluative conditioning technique) and an unrelated drawing task. The material we use is counter-stereotypes scenario material for older adults (Older adults’ counter-stereotypes video material, video material from “Dream Rider,” a commercial based on a true story by Taiwan Public Bank, 3min long). Thirty subjects were recruited to use the PANAS-X Positive Affect Self-Assessment Scale (Chinese version) (The Positive Affect Scale, PAS) to rate the pleasantness, arousal, and intensity of positive affect of the video using a Likert 9-point scale ranging from 1 (almost none) to 9 (very much) (see Appendix 3). The results of the subjects’ evaluations of the videos indicate that the videos are good at evoking positive impressions of the subjects about the older adults (See Table 3).

**Table 3:** Evaluation results for video materials.

Valence *M*(*SD*)	Arousal *M*(*SD*)	Positive emotional intensity *M*(*SD*)
6.37(1.56)	7.23(1.19)	6.83(1.39)

^a^*N*=30.

#### Design and Procedure

A 2 (group: experimental group [dual-task], control group [single-task])×3 (measurement time: pretest [IAT_1_], posttest [IAT_2_], intervention effect tracking test [IAT_3_ after 72h]) mixed design was used, and the dependent variable was the effect value (D) after the average reaction time before and after the IAT was converted *via* the Greenwald et al. (2003) method.

The participants were randomly assigned to either the experimental group or the control group, and the test time was arranged according to the different groups after the allocation. Each participant completed a practice experiment before starting the formal test, which lasted for 2min, and the formal test began after all the participants finished the practice experiment and confirmed they understood the experimental procedure.

**Figure 4:**
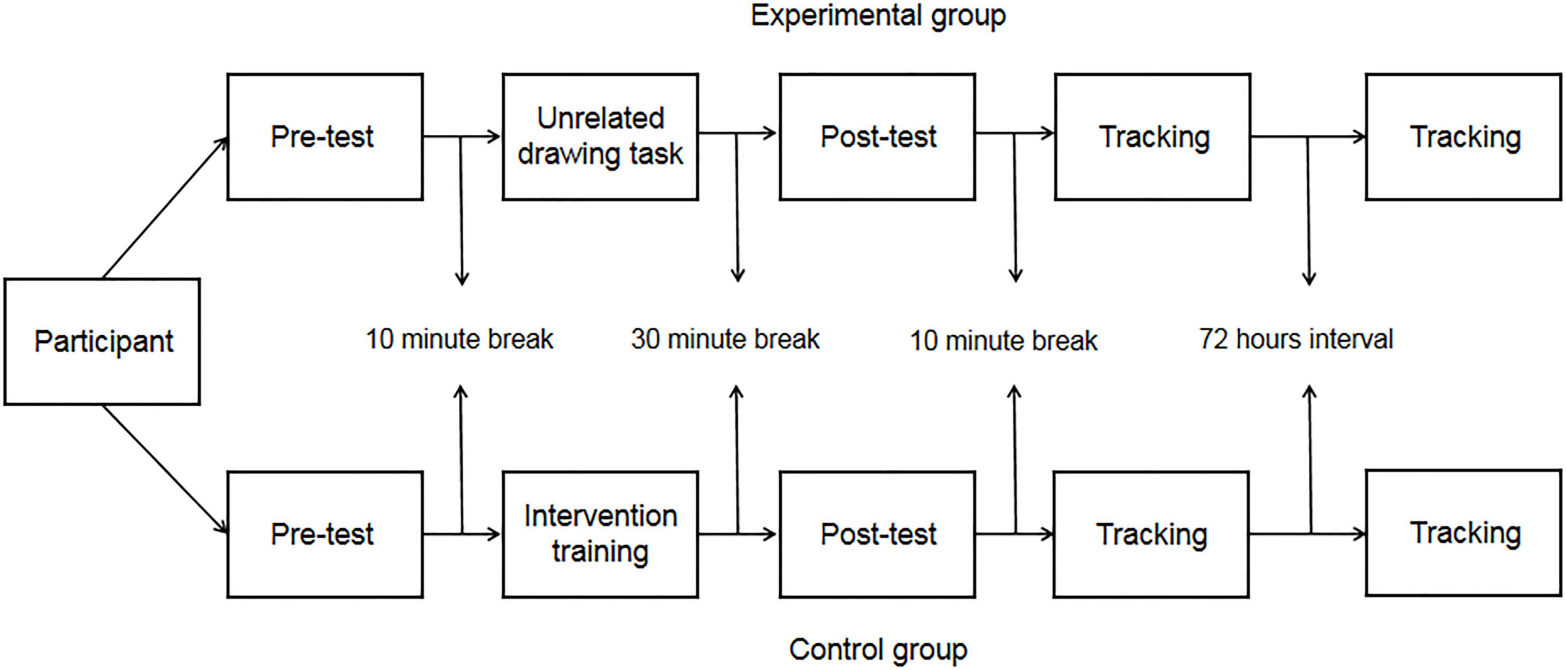
Experimental flow chart of Experiment 2a.

During the formal test, the tasks performed by the experimental group and the control group were different. The experimental group was required to complete pretest (IAT_1_), intervention training task, video task, posttest (IAT_2_), and tracking test (IAT_3_). The control group was required to complete the pretest (IAT_1_), intervention training task, unrelated drawing task, posttest (IAT_2_), and tracking test (IAT_3_). The pretest confirmed the baseline value, and the interval of the tracking test was 72h. See Figure 4 for the specific schedule of the experiment. After watching the video (counter-stereotypes scenario story), the experimental group was required to answer two questions related to the video (such as: 1. What kind of story does the video mainly tell? and 2. What do you think of your grandparents after watching the video?).

#### Results and Analysis

The data processing method of Experiment 2a was the same as that of Experiment 1, both of which were processed by the D-value calculation method of Greenwald et al. (2003). After the data processing of Experiment 2a, 64 valid data samples were obtained (experimental group 34, control group 30).

A repeated measures ANOVA showed that the main effect of measurement time was significant, *F*(2,124)=22.07, *p*<0.001, $\eta_{p}{}^{2}$ =0.26. The multiple comparison results showed that in the control group, there was a significant difference between the pretest and posttest effect values, *t* (29)=2.50, *p*=0.018, *Cohen’s d=1.67* (*M*_c-pre_=0.75, *SD*=0.35, *M*_c-post_=0.53, *SD*=0.23). There was no significant difference between the tracking test effect value and the posttest effect value in the control group, *t*(29)=−0.42, *p*=0.68, *Cohen’s d=−*0.16 (*M*_c-tra_=0.58, *SD*=0.35, *M_c-post_*=0.53, *SD*=0.23). The difference between the pretest and the tracking test effect values in the control group was significant, *t*(29)=2.15, *p*=0.04, *Cohen’s d=0.80* (*M*_c-pre_=0.75, *SD*=0.35, *M*_c-tra_=0.58, *SD*=0.35).

There was significant difference in the effect value of the experimental group between the pretest and posttest, *t*(33)=4.22, *p*<0.001, *Cohen’s d=1.47* (*M*_e-pre_=0.84, *SD*=0.37, *M*_e-post_=0.50, *SD*=0.31). There was also a significant difference between the tracking and the posttest effect values of the experimental group, *t* (33)=3.59, *p*<0.001, *Cohen’s d*= 1.25 (*M_e-post_*=0.50, *SD*=0.31, *M*_*e*-tra_=0.27, *SD*=0.20). The effect values of the pretest and tracking test in the experimental group were significantly different, *t*(33)=7.24, *p*<0.001, *Cohen’s d=2.52*(*M*_e-pre_=0.84, *SD*=0.37, *M*_e-tra_=0.27, *SD*=0.20) (see Table 4).

**Table 4:** Mean IAT effect values (D) by group.

Group^a^	Pretest *M(SD)*	Posttest *M(SD)*	Tracking *M(SD)*
Control group	0.75(0.35)	0.53(0.23)	0.58(0.35)
Experimental group	0.84(0.37)	0.50(0.31)	0.27(0.20)

a *N*=64.

**Figure 5:**
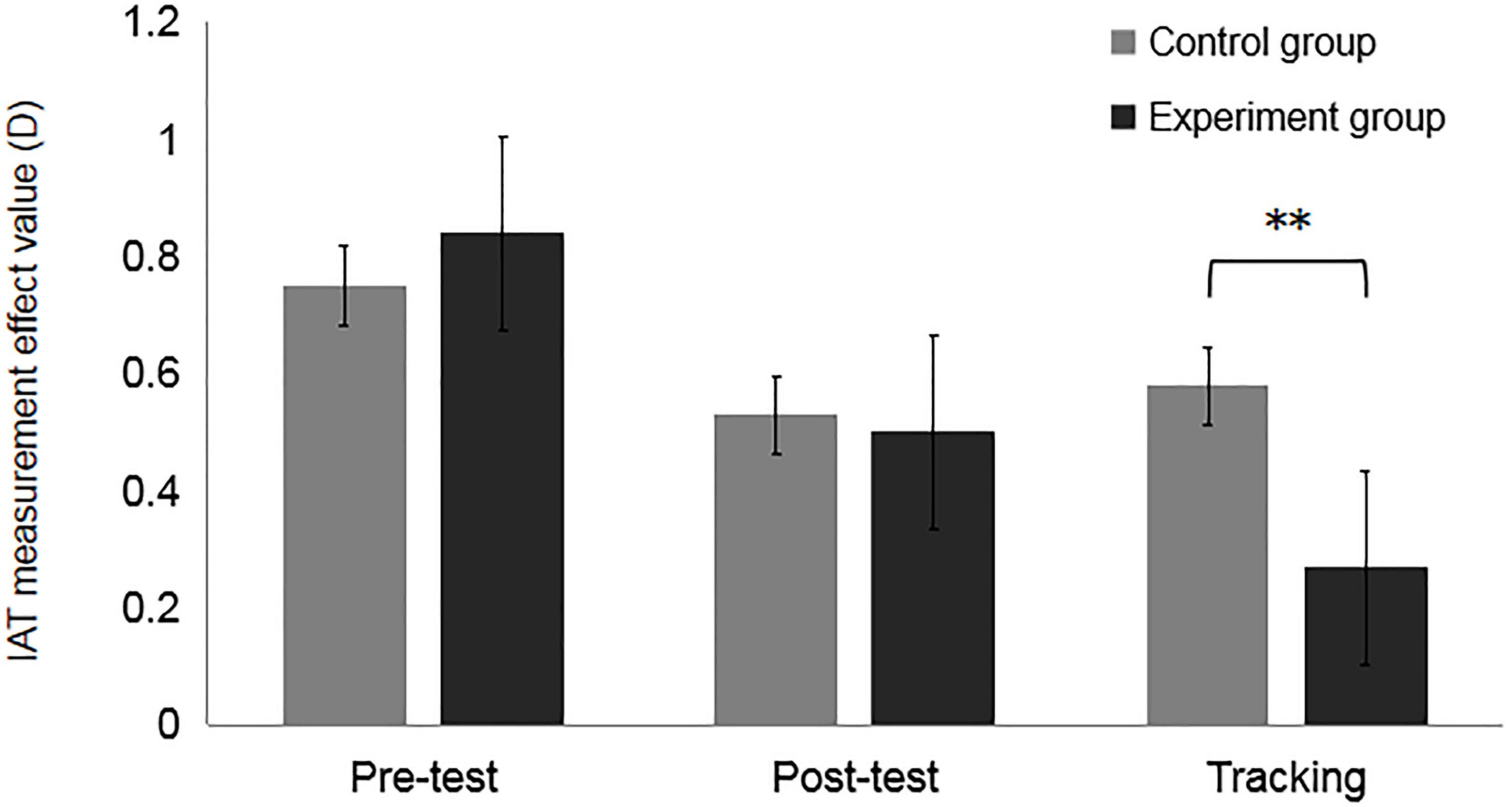
Results of the measurement of the effect value (D) of the training effect for different groups, **p<0.01.

The interaction between group and measurement time was significant, *F* (2, 124)=6.22, *p*=0.003, $\eta_{p}{}^{2}$ =0.09. Simple effects analysis showed that the effect values of the experimental group pretest were slightly larger than the effect values of the control group pretest in the group and measurement time pretest conditions, and there was no too significant difference between the effect values of the two groups pretest, *F*(1,62)=1.00, *p*=0.32, $\eta_{p}{}^{2}$ =0.02(*M_e-pre_*=0.84, *SD*=0.37, *M_c-pre_*=0.75, *SD*=0.35), indicating that there were no differences in subjects between the two groups. In the group and measurement time posttest conditions, the control group posttest effect values were slightly larger than the experimental group posttest effect values, and there was no significant difference between the two groups, *F*(1,62)=0.15, *p*=0.70, $\eta_{p}{}^{2}$ =0.02 (*M_c-post_*=0.53, *SD*=0.23, *M_e-post_*=0.50, *SD*=0.31). Under the conditions of group and measurement time tracking test, the effect values of the control group tracking test were significantly larger than the effect values of the experimental group tracking test, and the effect values of the experimental and control group tracking tests were significantly different, *F*(1,62)=18.55, *p*<0.001, $\eta_{p}{}^{2}$ =0.23 (*M*_*e*-tra_=0.27, *SD*=0.20, *M*_c-tra_=0.58, *SD*=0.35). This indicates that the effects of the intervention training were better maintained in the experimental group (See Figure 5).

### Discussion

The results of Experiment 2a found no significant difference between the experimental and control groups for both posttest results (see Figure 5). However, the results of the experimental and control groups differed significantly between the pretest and posttest after the intervention training, which was the same as the results of Experiment 1. The results of the follow-up test after 72h showed significant differences between the experimental and control groups. The results of the experimental group tracking test differed significantly from those of the posttest, but the results of the control group tracking test did not differ significantly from those of the posttest, and the results of the control group tracking test indicated that the effects of the intervention training were declining (see Figure 5).

The analysis of the results of Experiment 2a revealed that the retention effect of the intervention training could be effectively improved by increasing the training task and selecting a group of subjects with higher cognitive plasticity, and the retention effect of the intervention training could be effectively promoted by using the counter-stereotypes scenario story method.

## Experiment 2B: Intervention Training Effect Maintenance Tracking Test

In Experiments 1 and 2a, we conducted a tracking test comparison between two intervention methods. Experiment 2b adopted the same experimental design as Experiment 2a. The intervention training was conducted 72h after the completion of the pretest and posttest to ascertain which group’s intervention training effects lasted longer. In previous studies conducted in other countries, intervention training effects were shown to be maintained for a few hours or days at most (Lai et al., 2016; Burns et al., 2017). However, this previous research did not specify how many days the intervention effects lasted. Therefore, on the basis of previous studies, Experiment 2b explored whether the effect of intervention training could be effectively maintained for several days after increasing intervention training tasks and training times.

### Method

#### Participants

Seventy students (*M_age_*=12.15, *SD*=0.36) participated in Experiment 2b. All participants were randomly recruited from class six to class seven in the seventh grade of a middle school in Zunyi, Guizhou province, and were 12–13years old. Two participants did not complete all the tests because the speed of the test was too slow. Two participants failed to complete all the experimental tests due to computer error and two participated in the pretest but did not complete the tracking. After D-value treatment, it was found that the response errors of three participants exceeded 70%, so 61 effective samples were finally obtained (31 in the experimental group and 30 in the control group). All the recruited participants had normal or corrected-to-normal vision, were proficient in using computers, and had not participated in a similar experiment before. All participants volunteered and were given a small gift at the end of the experiment.

#### Materials

The experimental materials and measuring tools are the same as those in Experiment 2a.

#### Design and Procedure

The experimental materials and measurement tools were the same as those used in Experiment 2a. A 2 (group: experimental group [dual training tasks], control group [single training task])×3 (measurement time: pretest [IAT_1_], posttest [IAT_2_], Intervention Effect Tracking Test (IAT_3_) (after the second intervention training, 72h apart) mixed design with the dependent variable being the mean response time before and after the IAT test transformed by Greenwald et al. (2003) method of treatment for the effect value D-value.

**Figure 6:**
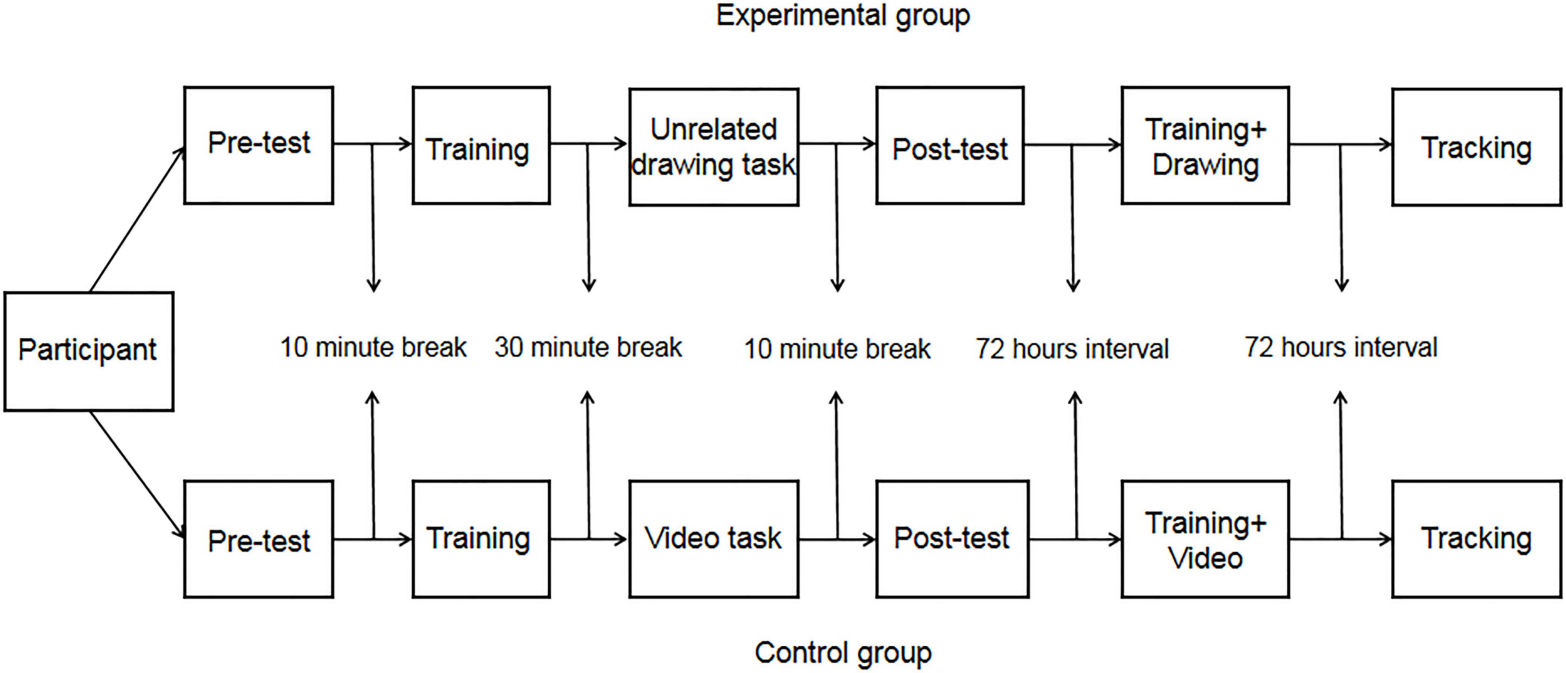
Experimental flow chart of Experiment 2b.

The subjects were randomly assigned to the experimental and control groups, and the test time was arranged according to the different groups after the assignment was completed, and the specific schedule of the experiment is shown in Figure 6. Before the formal experiment, each subject completed a practice experiment, which lasted 2min, until the subject indicated that they had fully understood the procedure and then the formal experiment began.

In the formal experiment, the experimental group completed the dual training tasks, while the control group completed single training tasks (same as Experiment 2a). In addition, the experimental group and the control group were required to repeat the training task and video/irrelevant drawing task 72h after the completion of the posttest (IAT_2_), followed by the tracking test 72h later (IAT_3_). See Figure 6 for the specific schedule of the experiment. After completing the video task (counter-stereotypes scenario) for the first time, the participants in the experimental group were asked the same questions as in Experiment 2a. After completing the video task (counter-stereotypes scenario) for the second time, the participants in the experimental group were asked different questions (such as 1. What is the average age of the older adults in the video? and 2. What do the older adults in the video do?).

#### Results and Analysis

The data processing method of Experiment 2b was the same as that of Experiment 2a, with the D-value calculation method proposed by Greenwald et al. (2003). After processing the data of Experiment 2b, we obtained 61 valid data samples (experimental group 31 and control group 30).

Repeated measures ANOVA showed that the main effect of measurement time was significant, *F*(2,118)=13.25, *p*<0.001, $\eta_{p}{}^{2}$ =0.18. The multiple comparison results showed that in the control group, the effect value of the pretest was significantly different from that of the posttest, *t* (29)=2.50, *p*=0.018, *Cohen’s d=0.93* (*M_c-pre_*=0.85, *SD*=0.46, *M_c-post_*=0.62, *SD*=0.32). There was no significant difference between the tracking test effect value and the posttest effect value in the control group, *t*(29)=−1.44, *p*=0.16, *Cohen’s d*=−0.53. There was also no significant difference between the pretest effect value and the tracking test effect value in the control group, *t*(29)=1.00, *p*=0.32, *Cohen’s d*= 0.37. There was a significant difference between the pretest and posttest effect values in the experimental group, *t*(30)=3.30, *p*= 0.002, *Cohen’s d*= 1.20 (*M_e-pre_*=0.88, *SD*=0.38, *M_e-post_*=0.55, *SD*=0.35). There was a significant difference between the tracking effect value and the posttest effect value in the experimental group, *t*(30)=2.16, *p<* 0.001, *Cohen’s d*= 0.79 (*M*_*e*-tra_=0.38, *SD*=0.29, *M_e-post_*=0.55, *SD*=0.35). There was also a significant difference between the pretest effect value and the tracking effect value in the experimental group, *t*(30)=5.68, *p<* 0.001, *Cohen’s d*= 2.07 (*M_e-pre_*=0.88, *SD*=0.38, *M*_*e*-tra_=0.38, *SD*=0.29). (see Table 5).

**Table 5:** Mean IAT effect values (D) by groups.

Group^a^	Pretest *M(SD)*	Posttest *M(SD)*	Tracking *M(SD)*
Control group	0.85(0.46)	0.62(0.32)	0.75(0.30)
Experimental group	0.88(0.38)	0.55(0.35)	0.38(0.29)

a *N*=61.

**Figure 7:**
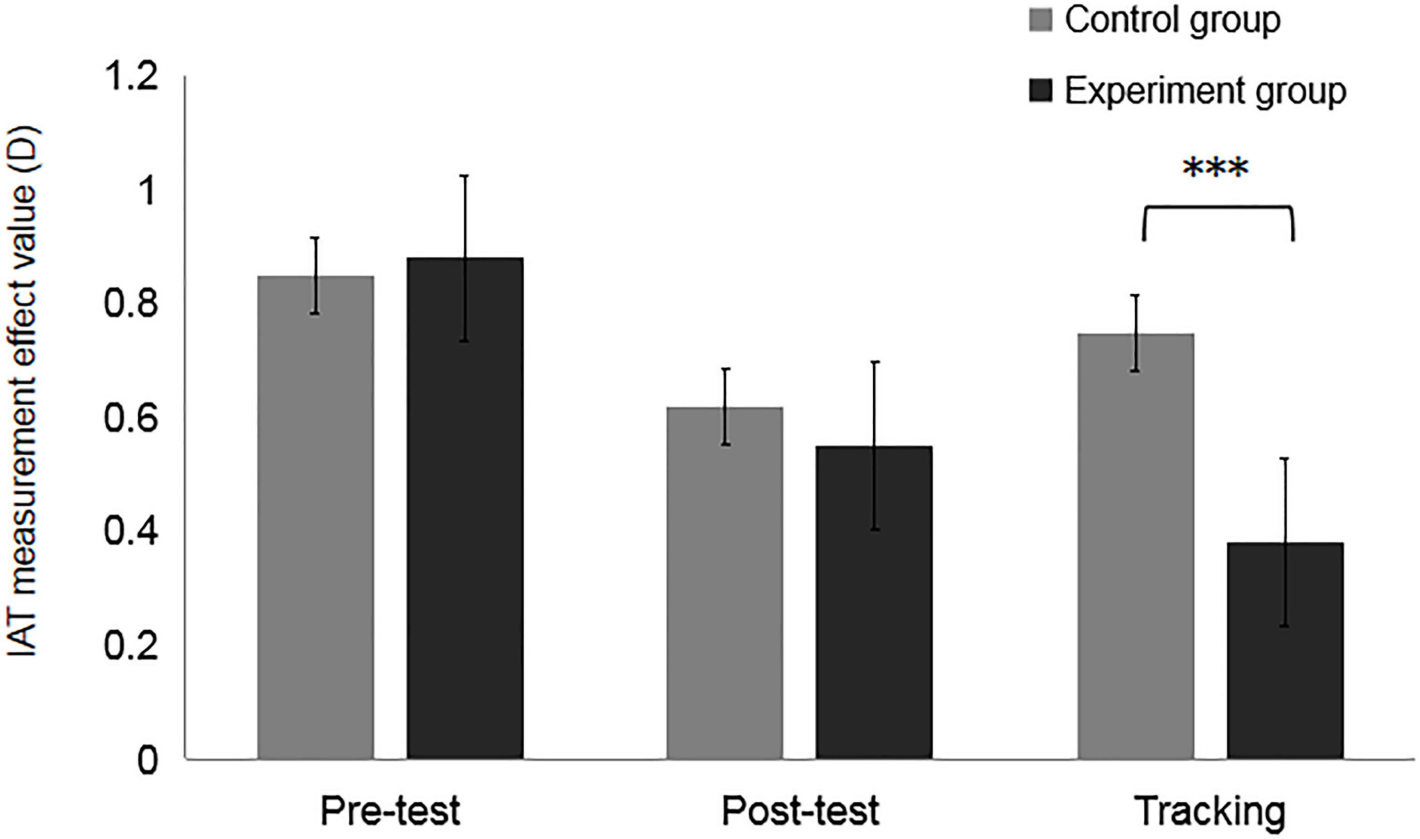
Results of the measurement of the effect value of training effect (D) for different groups, ***p<0.05.

The interaction between group and measurement time was significant, *F*(2, 118)=4.93, *p*=0.009, $\eta_{p}{}^{2}$ =0.07. Simple effects analysis showed that the effect values of the experimental group pretest were slightly larger than those of the control group pretest under the group and measurement time pretest conditions, and there was no significant difference between the effect values of the experimental group pretest and the control group pretest, *F*(1, 59)=0.07, *p*= 0.79, $\eta_{p}{}^{2}$ =0.001 (*M_e-pre_*=0.88, *SD*=0.38, *M_c-pre_*=0.85, *SD*=0.46). In the group and measurement time posttest conditions, the effect values in the control group posttest were slightly larger than those in the experimental group posttest, and the results of the experimental and control group posttests showed no too significant differences between the two groups, *F*(1, 59)=0.59, *p*= 0.45, $\eta_{p}{}^{2}$ =0.01 (*M_e-post_*=0.55, *SD*=0.35, *M_c-post_*=0.62, *SD*=0.32). Under the conditions of group and measurement time tracking tests, the effect values of the control group tracking tests were significantly larger than those of the experimental group tracking tests, and the results of the experimental and control group tracking tests differed significantly, *F*(1, 59)=22.82, *p<* 0.001, $\eta_{p}{}^{2}$ =0.28 (*M*_*e*-tra_=0.38, *SD*=0.29, *M*_c-tra_=0.75, *SD*=0.30). It shows that the experimental group has better retention of the training effect and can effectively reach 6days (See Figure 7).

### Discussion

Experiment 2b further compared the study methods on the basis of Experiment 2a to test whether the effects of the intervention training could be maintained for a longer period. We therefore increased the number of training tasks for the experimental group (multiple tasks) and the control group (single tasks) based on Experiment 2a. The results of Experiment 2b found no significant difference between the experimental and control groups on the pre- and postmeasures, a result consistent with Experiment 2a, that is, there was no immediate effect. However, after the addition of the training task, the results of the comparison between the two tracking tests and their pretest revealed that the comparison was not significant for the control group, while the opposite was true for the experimental group, indicating that the experimental group had better retention of the intervention training, which lasted for 6days, while the control group had poorer retention and faded after 72h.

## General Discussion

The purpose of this study was to investigate the effect of counter-stereotypes cognitive intervention training on aging stereotypes in 12- to 13-year-old adolescents. Three experiments were conducted to verify whether counter-stereotypes cognitive intervention training could effectively weaken adolescents’ aging stereotypes and whether the training effects could be maintained for a longer period by increasing the number of intervention training tasks and the number of training sessions.

The results of Experiment 1 showed that the subjects in the experimental group effectively weakened their age stereotypes through the intervention training compared to the control group. Experiment 1 was designed to validate the results of previous studies and also to verify the effect of age factor on the effect of intervention training. Levy et al. (2009) suggested that the formation of age stereotypes begins in childhood and continues to be internalized as we age, remaining in place until old age. Gonzalez et al. (2017) conducted intervention training with adolescent children aged 5–13years and showed that children aged 10years and older were most prominently affected by the intervention training. Therefore, the subject group recruited in this study was adolescents between 12 and 13years of age, and the effect retention effect of the intervention training was consistent with the results of previous studies using adult subjects, that is, the effect of performing a single intervention training was maintained for a few hours or days at most. Such results suggest that although adolescents are indeed better than adults in terms of cognitive plasticity, it is difficult to effectively promote the maintenance of intervention training effects after only a simple and limited number of training sessions, and therefore, further improvements should be made in the intervention training methods and the number of intervention training sessions.

Experiment 2a further explored the effect of counter-stereotypes intervention training on age stereotypes on the basis of Experiment 1. Comparing previous studies, it was found that the effect of counter-stereotypes intervention training had better effect in the short term, but the effect of intervention training was poorly maintained, which may be related to the single training method used in previous studies. Therefore, in Experiment 2a, we added a new intervention training task and also tested whether the addition of the intervention training task and the use of the new intervention training modality could effectively promote the maintenance of the intervention training effect. The results of the study showed that while there was no significant difference between the experimental and control groups on the posttest, there was a significant difference in the results of the follow-up test after 72h. That is, multiple training tasks are more conducive to the maintenance of intervention effects compared to single-task intervention training. This may be related to the fact that the subjects we recruited developed positive emotions toward the material in the experiment. Most of the subjects recruited for the study were left-behind children raised by older adults, whose emotional experiences led the subjects to be more likely to have positive emotions about the materials in the experiment. In contrast, the use of text plus video in the multiple training produced better positive effects than that produced by the single training, so that the results of the follow-up test after 72h differed significantly between the control and experimental groups. However, due to the high number of training tasks on that day, the fatigue effect caused the effect of the posttest not to be fully reflected.

Experiment 2b further validated the specific retention effect of intervention training by increasing the number of intervention training sessions on the basis of Experiment 2a. The results showed that there was no difference between the pre- and posttest results of the experimental and control groups. However, the results of the follow-up test showed a significant difference between the experimental and control groups after a second intervention training and again after an interval of 72h. The results of the comparison between the two groups of the follow-up test and their pretests revealed that the control group did not have significant comparison results, while the opposite was true for the experimental group. This suggests that using a single training task and simply increasing the number of intervention sessions is not good enough to improve the retention of training effects. The retention effect of the intervention training in the experimental group was maintained for 72h, mainly because the intervention training task used was a combination of the intervention training tasks used in previous studies, and a new task, counter-stereotypes scenarios, was added to the previous intervention training tasks. The use of video for intervention training allows subjects to feel more engaged and can positively influence them from multiple channels (Dasgupta and Greenwald, 2001; Marini et al., 2012; Meijs et al., 2015). On the other hand, the counter-stereotypes scenario uses video material related to the positive aspects of older adults, and the subjects have already associated the positive words in the intervention training material with older adults when completing the intervention training task, and after continuous matching and responding, the subjects have formed relevant links. And the video material used coincided with the positive aspects of older adults, so that subjects maintained better effects on the training task after completing all tasks even though the interval was longer than when only a single task was used in the control group.

The theoretical contributions of this study are mainly in the following areas. First, this study found that the tracking test results of multiple training methods differed significantly from those of single training methods, meaning that multiple training methods performed better than single training methods in terms of retention of training effects. This finding complements previous research on counter-stereotypes intervention training to alleviate aging stereotypes and further explores the best training modality to intervene with aging stereotypes. Second, the results of this study demonstrated that the counter-stereotypes intervention was also effective for adolescents aged 12 to 13years, which expands the age range of the counter-stereotypes intervention.

This paper also provides some practical implications by verifying the effect of counter-stereotypes intervention training on age stereotypes and the retention effect of the intervention training effect. Combined with the results of this study, we can use the intervention method similar to counter-stereotypes scenario story method in our daily life to carry out unconscious intervention and interstitial reinforcement, such as using TV and online media, broadcasting some representative public service announcements, or offering some related activities in the seventh grade of secondary school, so as to enhance the public’s understanding of age stereotypes. At the same time, regions that have the conditions can organize regular activities for children of 12- to 13-year-olds to care for the older adults, such as regular visits to older adults’ homes or going into the community to accompany the older adults.

Although the present study provides further evidence for research related to counter-stereotypes cognitive training in mitigating aging stereotypes, some gaps remain in this study and future research could be conducted in the following areas. First, the sample size of the subjects was relatively small, concentrated in remote areas, and some of the samples were left-behind children, so the sample was not representative enough. Therefore, the next study could expand the validity and generalizability of the results by selecting adolescents from different regions to test the sample size. Second, in the IAT measurement process, both the control group and the experimental group need to conduct pre and posttests as well as follow-up tests, so there is a practice effect. Although all the trials in our experimental measurement procedure are presented randomly, there is still a certain practice effect, and we also found that too many trials in the IAT can cause subject fatigue and thus make the measurement results inaccurate, so the duration of the measurement procedure should be adjusted appropriately in future studies. Third, this study did not include data from adults, and follow-up studies should add adult subjects as a reference group to further verify the effectiveness of counter-stereotypes cognitive training in mitigating aging stereotypes in child subjects. Fourth, the arousal of the video material in Experiment 2a and 2b was 7.23, which may be significantly higher than the median value (5), and the arousal test of the video material in the follow-up study should add a pre- and posttest of subjects’ emotions to test whether the effect of counter-stereotypes stories is caused by emotional arousal.

## Conclusion

The study explored the effects of counter-stereotypes cognitive training on the effects of aging stereotype in adolescents and compared the effects of thee dual and single intervention training on the aging stereotype and its maintenance effect. The results showed that evaluative conditioning can effectively weaken aging stereotypes in 12- to 13-year-olds. However, the retention effect was poor. Compared with a single training task, the effect of a dual-task was better, and the intervention effect was maintained for longer when the training frequency of the dual-task was increased.

## Data Availability Statement

The original contributions presented in the study are included in the article/supplementary material, further inquiries can be directed to the corresponding author.

## Ethics Statement

Written informed consent was obtained from the individual(s), and minor(s)’ legal guardian/next of kin, for the publication of any potentially identifiable images or data included in this article.

## Author Contributions

LC, XZ, SF, LF, and JZ: conceptualization. XZ: methodology, data analysis, and writing original draft preparation. JZ and XZ: software. LC, XZ, and LF: validation. XZ and LF: investigation. LF: original draft confirmation and submission. SF: write and revise all review comments, refine theory and data supplementation, and submit final version of manuscript. LC: project administration. All authors contributed to the article and approved the submitted version.

## Funding

This work was supported by the Gansu Province Philosophy and Social Science Planning Office under Grant [Project no. YB044]; Northwest Normal University under grant [no. 2018SKGG06]; and Northwest Normal University under grant [no. NWNU-LKQN-18-36].

## Conflict of Interest

The authors declare that the research was conducted in the absence of any commercial or financial relationships that could be construed as a potential conflict of interest.

## Publisher’s Note

All claims expressed in this article are solely those of the authors and do not necessarily represent those of their affiliated organizations, or those of the publisher, the editors and the reviewers. Any product that may be evaluated in this article, or claim that may be made by its manufacturer, is not guaranteed or endorsed by the publisher.
